# Effects of Salinity on Bacterial Spot Disease, Physiology, Growth, Fruit Quality, and Transcriptomic Responses in Tomato Plants

**DOI:** 10.3390/plants15162457

**Published:** 2026-08-13

**Authors:** Ketsira Pierre, Ana I. Vargas, Geoffrey Meru, Bruce Schaffer, Jeffrey B. Jones, Shouan Zhang

**Affiliations:** 1 Tropical Research and Education Center, Department of Plant Pathology, University of Florida, IFAS, Homestead, FL 33031, USA; ketsirapierre@ufl.edu; 2 Department of Plant Pathology, University of Florida, IFAS, Gainesville, FL 32611, USA; jbjones@ufl.edu; 3 Tropical Research and Education Center, Department of Horticultural Sciences, University of Florida, IFAS, Homestead, FL 33031, USA; avargas22@ufl.edu (A.I.V.); gmeru@ufl.edu (G.M.); bas56@ufl.edu (B.S.)

**Keywords:** *Xanthomonas perforans*, tomato, bacterial spot, soil salinity, abiotic and biotic stress, transcriptomics

## Abstract

Soil salinity and bacterial spot of tomato (BST), caused by *Xanthomonas perforans*, are major abiotic and biotic stresses limiting tomato production, particularly in Florida. While their individual effects are well documented, the impact of soil salinity on BST has not yet been investigated. This greenhouse study evaluated how increasing irrigation water salinity (electrical conductivity [EC] = 0.5, 3, 5, or 7 dS m^−1^) affected tomato growth, physiology, BST severity, fruit quality, and transcriptomic responses. Salinity reduced plant growth and BST severity but did not directly affect *X. perforans* populations. Results indicated that reduced plant physiological activity (net CO_2_ assimilation [*A*], transpiration [*E*], and stomatal conductance [*g_s_*]) contributes to lower disease levels. Increased salinity led to more solute concentrations, altered sugar metabolism, and improved perceived taste, as supported by taste panel, osmolality, and transcriptomic analyses. They also showed that transcriptional responses to salinity (EC = 7 dS m^−1^) and *X. perforans* infection were strongly time-dependent. Salt-treated plants exhibited fewer differentially expressed genes following inoculation, whereas comparisons between EC 7-treated and control plants revealed extensive salinity-induced reprogramming. KEGG analysis indicated enrichment of photosynthesis, carbon metabolism, amino acid biosynthesis, and ribosome pathways, while defense-related pathways, including MAPK signaling and plant–pathogen interaction, were downregulated, suggesting that tomato prioritized adaptation to salinity over pathogen defense.

## 1. Introduction

Tomatoes are a staple food in cuisines worldwide. In the United States, tomato production is valued at $1.97 billion [1]. In Florida, tomatoes are the number one vegetable in terms of hectares harvested and total production. With an agricultural production value at over $330 million [2], tomatoes are a crucial part of Florida’s economy; therefore, their proper care is essential. Unfortunately, tomato production is frequently challenged by a combination of biotic and abiotic stressors. A major biotic stressor of tomato plants is bacterial spot of tomato disease (BST) [3], and a major abiotic stressor of tomato and many other crops is soil salinity [4,5]. This study aims to explore the impact of soil salinity on tomato plants and BST.

BST is a devastating bacterial disease primarily caused by *X. euvesicatoria* pv. *perforans* (referred to *X. perforans* thereafter) in Florida, although it is also caused by *X. vesicatoria*, *X. euvesicatoria* pv. *euvesicatoria*, and *X. hortorum* pv. *gardneri.* This disease significantly affects tomato production worldwide. Under greenhouse conditions, the pathogen is disseminated through water droplets aerosolized by overhead irrigation and is further spread by close plant spacing and handling of wet plants [6,7,8]. The pathogen enters plant tissue through natural openings and wounds, causing water-soaked small lesions initially and then brown to black lesions on leaf surfaces. Eventually these lesions spread throughout the plant, leading to yield loss and reduced fruit quality. In 2007–2008, it was reported that losses due to bacterial spot were over $7600 per hectare in Florida [9]. Additionally, in 2010, the Midwestern USA’s processing industry reported losses of $7–8 million due to BST [9]. Therefore, the management of BST remains an ongoing challenge for growers, primarily due to the pathogen’s resistance to copper bactericides and the lack of commercially available resistant tomato varieties.

Soil salinization, the accumulation of soluble salts in soils, typically occurs naturally, but is often exacerbated by human activities such as overfertilization, land clearing, and poor irrigation practices [4,5]. Also, in many agricultural regions including Florida, saltwater intrusion inland as a result of sea-level rise is increasing the salinity of the soil and irrigation water, thus threatening crop production [10]. According to FAO, soil salinization results in substantial losses in agricultural productivity worldwide [11]. Estimates reported in the literature vary, with FAO also citing global crop production losses from salt-induced land degradation in irrigated areas of approximately $27.3 billion annually [11,12]. Furthermore, it is projected that, by 2050, 50% of land suitable for growing crops will be impacted by salinity [4,13]. While small amounts of salts are essential for plant growth, salt levels beyond a crop’s tolerance can negatively affect its morphology, physiology, and productivity, ultimately leading to plant damage or death [4,14].

The impacts of salinity on disease vary based on the pathogen and crop. Soil salinity has been shown to enhance the proliferation of *Fusarium solani*, which causes wilt disease in potato, and angular leaf spot in cucumber caused by *Pseudomonas syringe* pv. *lachrymans* [15,16]. Conversely, in dragon fruit, salinity treatments reduced stem canker caused by *Neoscytalidium* sp. [17]. In tomatoes, salt treatments, depending on the concentration and type of salt (e.g., chloride, sulfate, carbonate salts of calcium, magnesium, and sodium) used, have been shown to both increase and reduce susceptibility of tomato plants to bacterial spot caused by *Xanthomas euvesicatoria* and gray mold incited by *Botrytis cinerea* [4,18]. Tomato is considered moderately tolerant to salinity, with yield reductions occurring above a threshold salinity level of approximately 2.5 dS m^−1^ [19]. Depending on the cultivar and crop condition, low to moderate salinity levels, approximately 25–50 mM NaCl (2.5–5.0 dS m^−1^), may enhance plant resilience by inducing adaptive responses, including reduced disease, increased antioxidant activity, and osmotic adjustment [20,21,22]. However, higher salinity levels, generally ≥75–100 mM NaCl (7.5–10 dS m^−1^), can impair plant performance by causing osmotic stress, ion toxicity, and oxidative damage, thereby increasing susceptibility to stress and certain diseases [4,21,22,23]. Therefore, salinity responses in crops may represent a potential trade-off between negative effects on plant growth and physiology, and positive effects on disease and crop productivity. Understanding this balance is particularly relevant in tomato production, where moderate salinity levels may simultaneously induce beneficial adaptive responses and impose physiological constraints.

Many studies on the effects of soil salinity in tomatoes focus on seed or early seedling stages in nutrient solutions [24,25,26,27]. In contrast, our study uniquely investigates this effect on four-week-old tomato plants grown in soils, more closely reflecting commercial tomato production. The objectives of this study were to evaluate the effects of soil salinity on tomato plant physiology, BST severity, plant growth and fruit quality, and to investigate changes in gene expression in response to combined effects of salinity and BST through comparative transcriptomic analysis. In this study, the salt sodium chloride (NaCl), was selected for its dominance and significance in agricultural soils, even though salinization can arise from a variety of salts [4].

## 2. Results

### 2.1. Impact of Salinity on Plant Growth and BST

**Figure 1:**
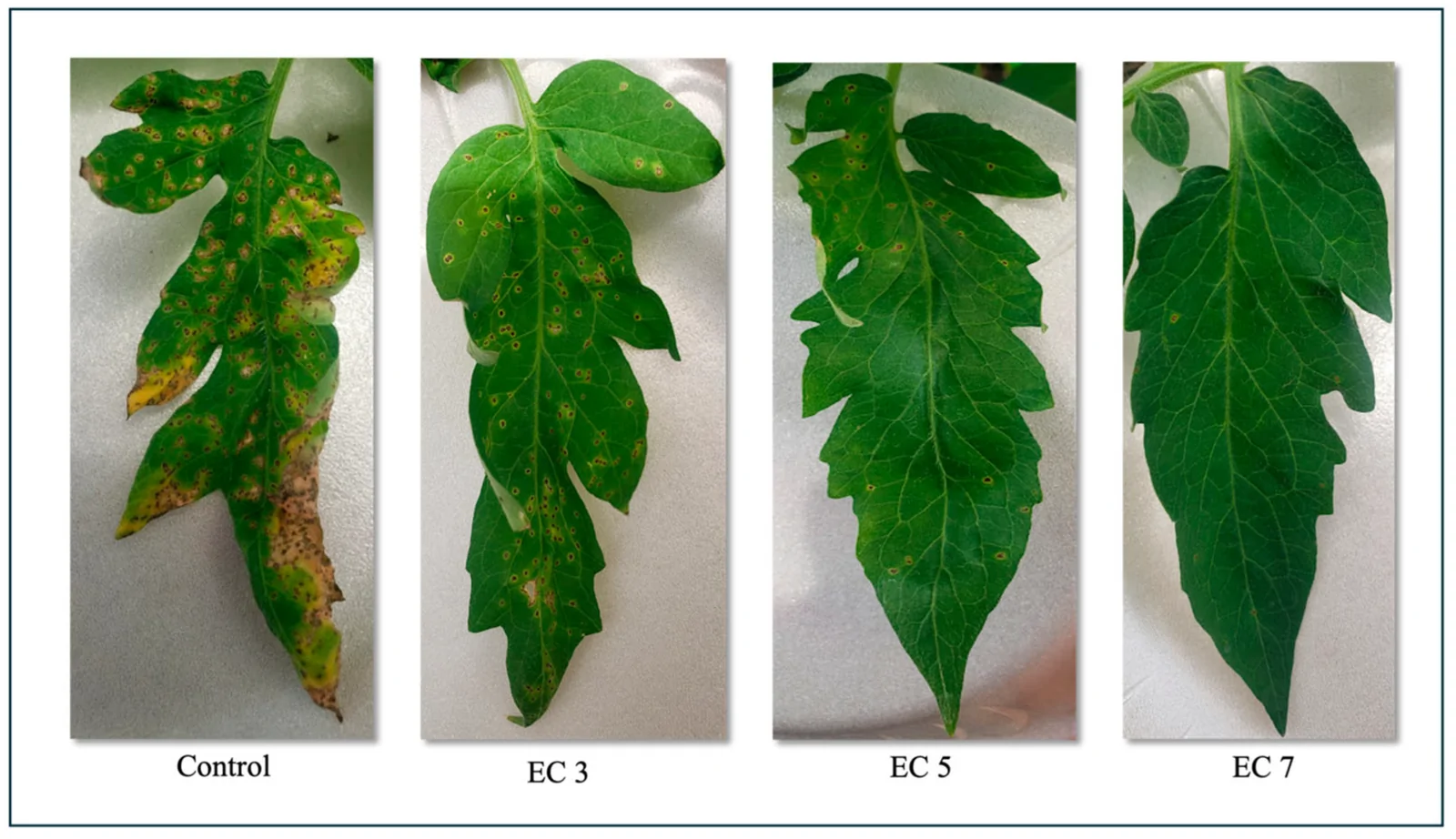
Disease severity of bacterial spot on tomato leaves inoculated with QL strain of *X. perforans* under salinity treatments in greenhouse. Photo courtesy of author Ketsira Pierre.

Salt-treated plants showed a clear reduction in BST disease symptoms (Figure 1). EC 3-treated plants had fewer lesions and minimal yellowing; EC 5-treated plants showed even fewer lesions and no yellowing; and EC 7-treated plants displayed only minimal lesions with no other visible BST symptoms. In contrast, control plants exhibited the most severe symptoms, including extensive lesion development, leaf yellowing and necrosis. Salinity treatments led to a significant reduction in disease severity (DS) and disease progression (AUDPC) compared to the control (Table 1). Among the inoculated, salt-treated plants showed a 79–95% decrease in DS and an 85–97% reduction in AUDPC compared to the control.

While salinity treatments reduced disease, there was a negative impact on plant growth. A significant interaction was observed between salt treatment and inoculation for fresh shoot weight, but not for plant height (Table S1), indicating that the effect of inoculation on biomass varied across salinity levels. Therefore, salinity responses are presented separately for inoculated and non-inoculated plants. Inoculated plants had a 0.58–12% decrease in plant height compared to the control. Fresh shoot weight was significantly reduced by 23% only in plants treated with EC 7, whereas no significant differences were observed in plants treated with EC 3 or EC 5 compared with the control. In non-inoculated plants, plant height did not differ significantly among treatments, although there was a general decline in height with increasing salinity. In contrast, fresh shoot weight decreased significantly by 17–26%, with the highest values observed in the control and the lowest in the EC 7 treatment. Inoculated plants had significantly lower fresh shoot weight than non-inoculated plants in the control, EC 5, and EC 7 treatments, but not in the EC 3-treatment (Table S2).

The EC values of water leachate and soil samples reflected the salt accumulation in salinity treatments (Table 2). Before treatment, water leachate collected at the beginning of the experiment showed similar EC values across all salinity treatments. By the end of the experiment, however, there was a sharp increase in EC related to salt concentration. Control samples showed the lowest EC, while EC 7 samples had the highest. A similar trend was observed in soil samples collected at the end of the experiment, with EC values increasing significantly with increasing salt levels. These results indicate significant differences in soil salinity in the treatments, which had a negative impact on overall plant growth, and also significantly decreased BST compared to the control.

### 2.2. Leaf Gas Exchange in Response to Salinity and BST

**Figure 2:**
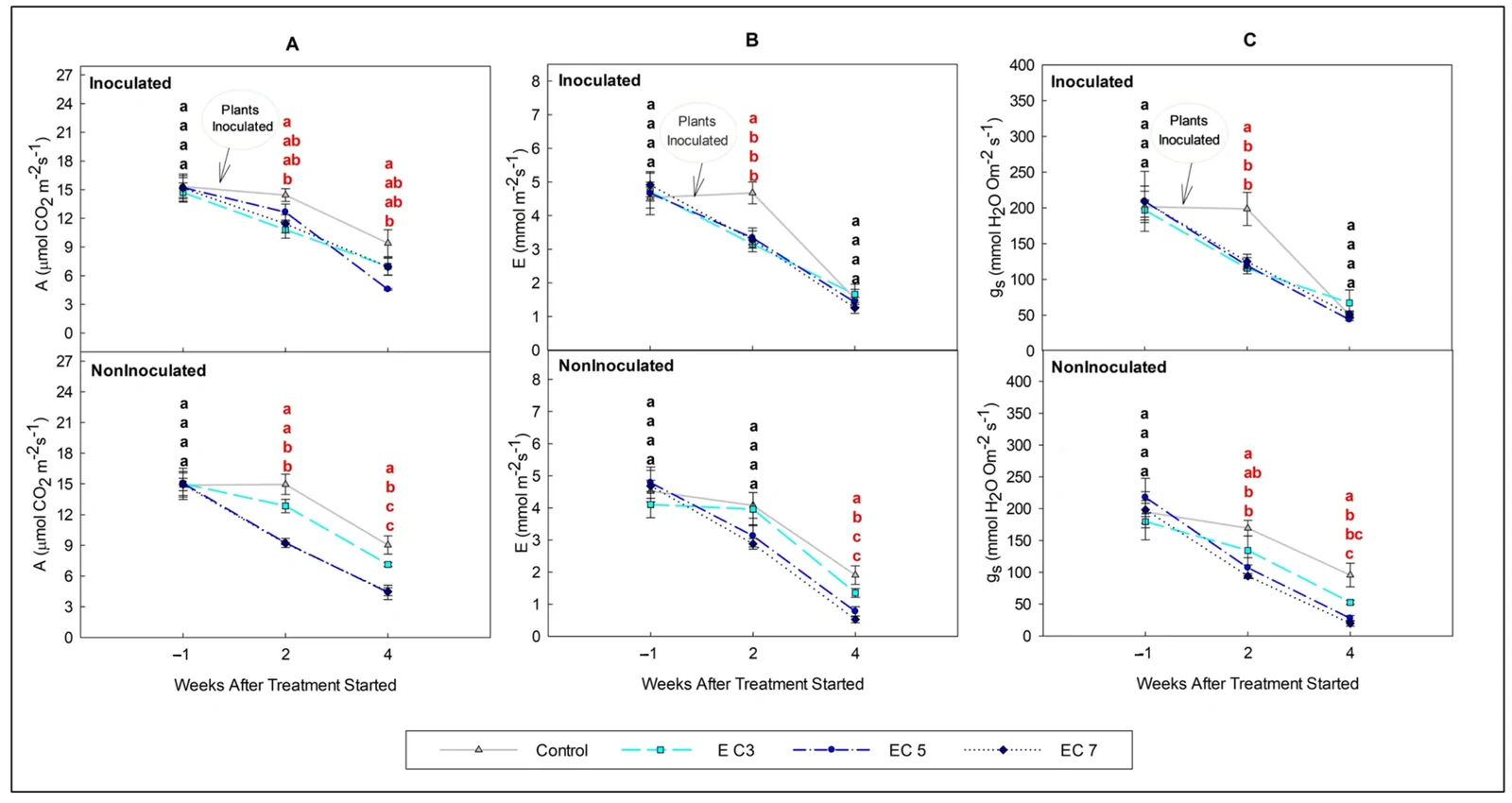
Effect of salinity treatment on gas exchange parameters. Measurements of (**A**) net CO_2_ assimilation (*A*), (**B**) transpiration (*E*), and (**C**) stomatal conductance (*g_s_*) to assess the physiological response of inoculated and non-inoculated tomato plants under salt stress. Each treatment included five replicates, and error bars represent STDEV. Statistical analysis was performed by ANOVA followed by Waller–Duncan k-ratio t - test; different color and letters above the bars indicate statistically significant differences among treatments (*p* < 0.05).

Overall, net CO_2_ assimilation (*A*), transpiration (*E*), and stomatal conductance (*g_s_*) declined over time in both inoculated and non-inoculated plants (Figure 2). Significant salinity × inoculation treatment interactions were detected for *A* and *g_s_* at specific timepoints, while there was only a marginal interaction for *E* (Table S3). Therefore, salinity responses were analyzed separately for inoculated and non-inoculated plants. Prior to salinity treatments (Week −1), there were no significant differences among treatments for *A*, *E* and *g_s_*.

After salinity treatments were initiated, control plants had consistently higher leaf gas exchange values than salt-treated plants. Two weeks after treatments started, salinity significantly affected net CO_2_ assimilation (*A*) in both inoculated and non-inoculated plants, with EC 7-treated plants having the lowest values compared with the control (Figure 2A). These differences were more pronounced in non-inoculated than inoculated plants. There was also a significant interaction between salinity and inoculation treatments, with inoculated plants showing higher *A* under EC 5 treatment compared with non-inoculated plants. In inoculated plants, transpiration also declined significantly in EC 3, 5 and 7 treatments compared to the control by Week 2 (Figure 2B). By Week 4, values had dropped across all treatments, reducing statistical separation. In non-inoculated plants, significant differences emerged by Week 4, with plants in the EC 5 and EC 7 treatments having the lowest transpiration rates.

For inoculated plants, there were differences in *g_s_* among salinity treatments at Week 2 (Figure 2C). However, treatment differences were not significant by Week 4 due to an overall decline in *g_s_* across all treatments. In non-inoculated plants, *g_s_* differed between the control and EC 5 and 7 treatments at Week 2, and among all salt treatments at Week 4, with greater reductions at higher salinity levels. Additionally, at Week 4, a significant salinity × inoculation interaction was observed for *g_s_* with inoculated and non-inoculated plants differing significantly under the control and EC 7 treatments. Overall, these results suggest that both salinity and disease lead to progressive physiological decline in gas exchange.

### 2.3. Effect of Salinity Treatment on X. perforans Populations In Vitro and In Planta

**Figure 3:**
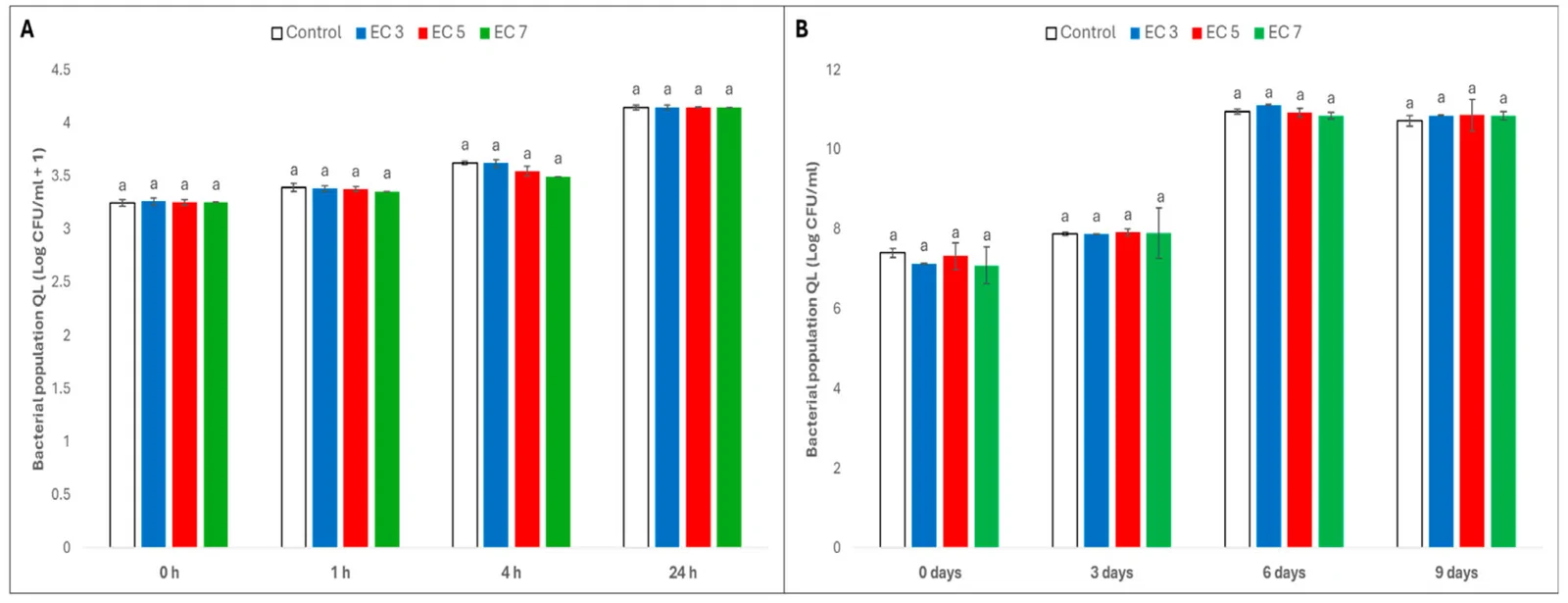
Effect of salinity treatments on bacterial populations. (**A**) In vitro activity of salinity treatments against *X. perforans* (QL) at 0 h, 1 h, 4 h, and 24 h. (**B**) In planta bacterial populations of *X. perforans* (QL) in tomato leaves via infiltration under salinity treatments at 0-, 3-, 6-, and 9 days post-inoculation. Each treatment included three replicates, and error bars represent STDEV. Statistical analysis was performed by ANOVA followed by Tukey’s post hoc test; same letters above the bars indicate no significant statistical differences among treatments (*p* ≥ 0.05).

Growth of *X. perforans*, both in vitro and in planta, showed no statistically significant differences across treatments at any given timepoint compared to the control (Figure 3). In vitro assays conducted over a 24 h period showed no statistical differences in bacterial populations in any treatment at any sampling time, compared to the control (Figure 3A). In all treatments, bacterial populations continued to steadily rise over time. Similarly, tomato leaves infiltrated with *X. perforans* also demonstrated no significant differences among salinity treatments at any given timepoint throughout the 9-day experimental period (Figure 3B). These findings suggest that salinity treatments did not have a direct effect on the growth of *X. perforans*.

### 2.4. Osmotic Adjustment in Tomato Plants Under Salinity Treatments

Osmolality (Osm) increased slightly as salinity treatment level increased, ranging from 0.32 mmol/kg in control plants to 0.36 mmol/kg in the EC 7 treatment; however, the differences were not statistically significant (Table 3). Similarly, osmotic potential tended to be more negative with increasing salinity, from −0.80 MPa in controls to −0.89 MPa in the EC 7 treatment, but the differences were also not significant. Osmotic adjustment increased with increasing salinity, ranging from 0.06 MPa in EC3 and EC5 to 0.10 MPa in EC 7 treatments, albeit not statistically significant among the treatments. Although the differences were not statistically significant, these results suggest a gradual shift toward increased osmotic regulation with increasing salinity.

### 2.5. Impact of Salinity on Fruit Flavor and Yield

**Figure 4:**
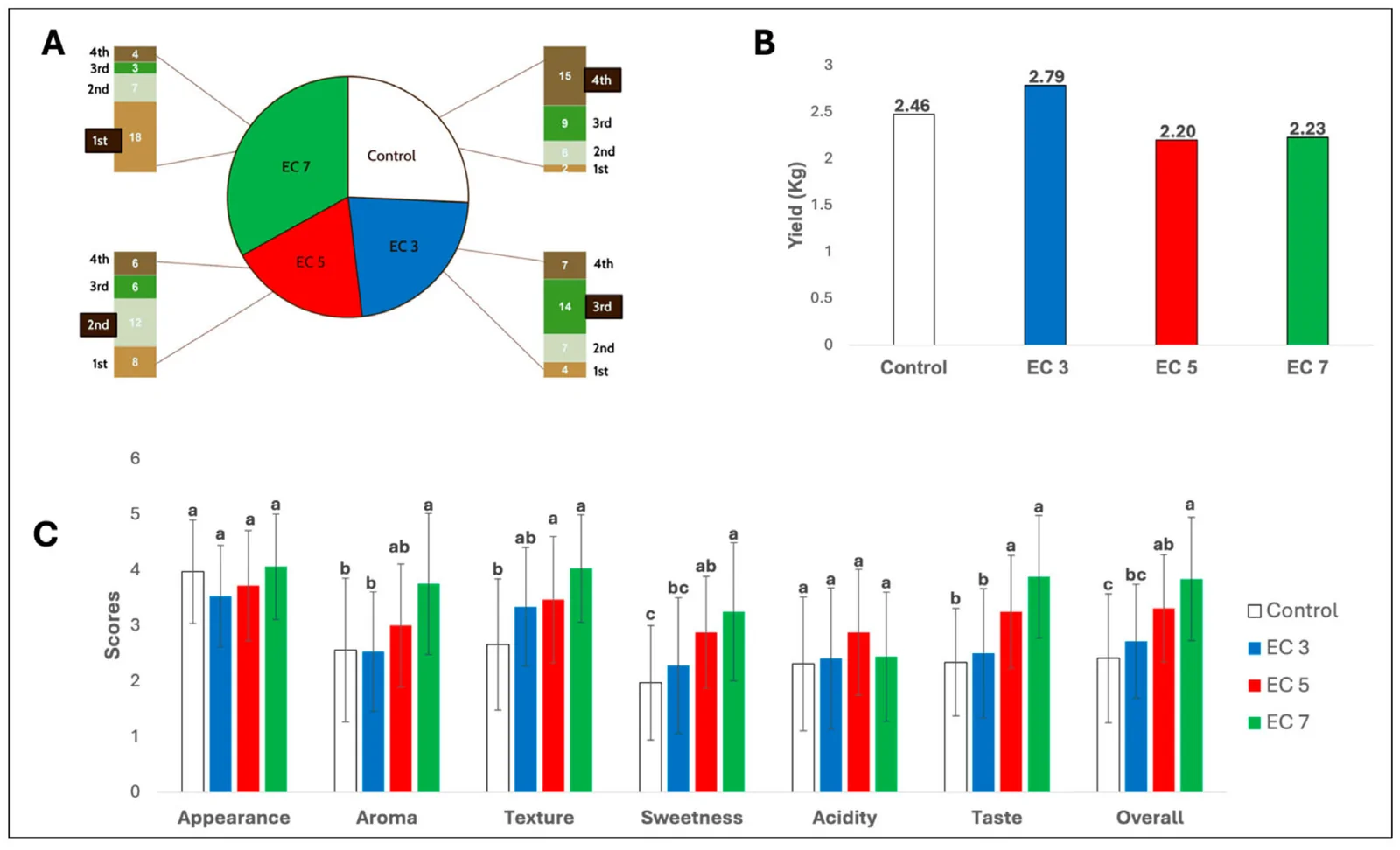
Results from the tomato taste panel of tomato fruit quality in each salinity treatment. (**A**) Participant rankings of overall taste preference for tomatoes grown under salinity treatments. (**B**) Total fruit yield (kg) of plants grown under salinity treatments. Yield data were pooled across replicates and are presented as the total fruit mass per treatment. (**C**) Participant evaluations of taste attributes for tomatoes grown under salinity treatments. A total of 32 participants rated texture, acidity, sweetness, and other taste attributes using a 5-point scale, where 5 = excellent and 1 = poor. Values represent the mean treatment scores for each attribute. Each treatment included three replicates, and error bars represent STDEV. Statistical analysis was performed by ANOVA followed by Tukey’s posthoc test; different letters above the bars indicate statistically significant differences among treatments (*p* ≤ 0.05).

Blind-taste panel participants preferred tomato fruit produced from plants treated with the salinity treatments than the control (Figure 4). The fruit from plants in the EC 7 treatment were the most favored, followed by those in the EC 5 and EC 3 treatments, and fruit from the control were the least favored (Figure 4A). Total fruit yield was highest in the EC 3-treated plants, followed by the control, EC 7, and the EC 5-treated plants (Figure 4B). Taste evaluations showed that salt-treated fruit scored higher across most attributes. However, appearance and acidity ratings did not differ significantly among treatments (Figure 4C). Fruit from the EC 7-treated plants were consistently rated the highest for attributes such as aroma, texture, sweetness, taste, and overall flavor, whereas fruit in the control treatment were rated lowest with significant differences among the treatments. Fruit in the EC 3 treatment were not significantly different from those in the control treatment and received similar ratings across all attributes. Fruit in the EC 5 and EC 7 treatments were preferred for aroma, texture, sweetness, taste, and overall quality.

### 2.6. Comparative Transcriptomic Analysis of Tomato Plants Under Salinity Treatment and BST

#### 2.6.1. Transcriptomics Assembly Statistics

Of the 57 samples, 53 RNA-seq libraries were constructed and sequenced. The number of raw reads per sample ranged from approximately 70.8 million to 158.3 million, generating between 10.62 Gb and 23.74 Gb of raw data (Table S4). After quality control, clean reads ranged from 68.1 million to 149.1 million, indicating a high retention rate. Sequencing quality had an average error rate of 0.01%, Q30 scores above 94%, and GC content ranging from 41.6% to 43.2%. Following alignment to the *Solanum lycopersicum* reference genome, an average of 88.7 million clean reads per sample were analyzed, with 93.5% mapping to the reference genome, 91.7% mapping uniquely, and 87.0% properly mapped. In total, 31,210 genes were annotated, providing a comprehensive and high-quality dataset suitable for downstream transcriptomic analyses.

#### 2.6.2. Transcriptomic Comparison of Inoculated EC 7 and Inoculated Control Plants

Consistent with the previous results, plants in the EC 7 treatment exhibited significant reduction in BST disease severity and AUDPC compared to the control (CK) (Table S5). Disease severity was about 24% in CK plants and 0.6% for EC 7-treated plants. AUDPC showed a similar trend with disease progressing significantly more in CK plants than in EC 7-treated plants. Additionally, there was a sharp increase in water leachate and soil EC in the EC 7-treated plants compared to the CK (Table S6).

**Figure 5:**
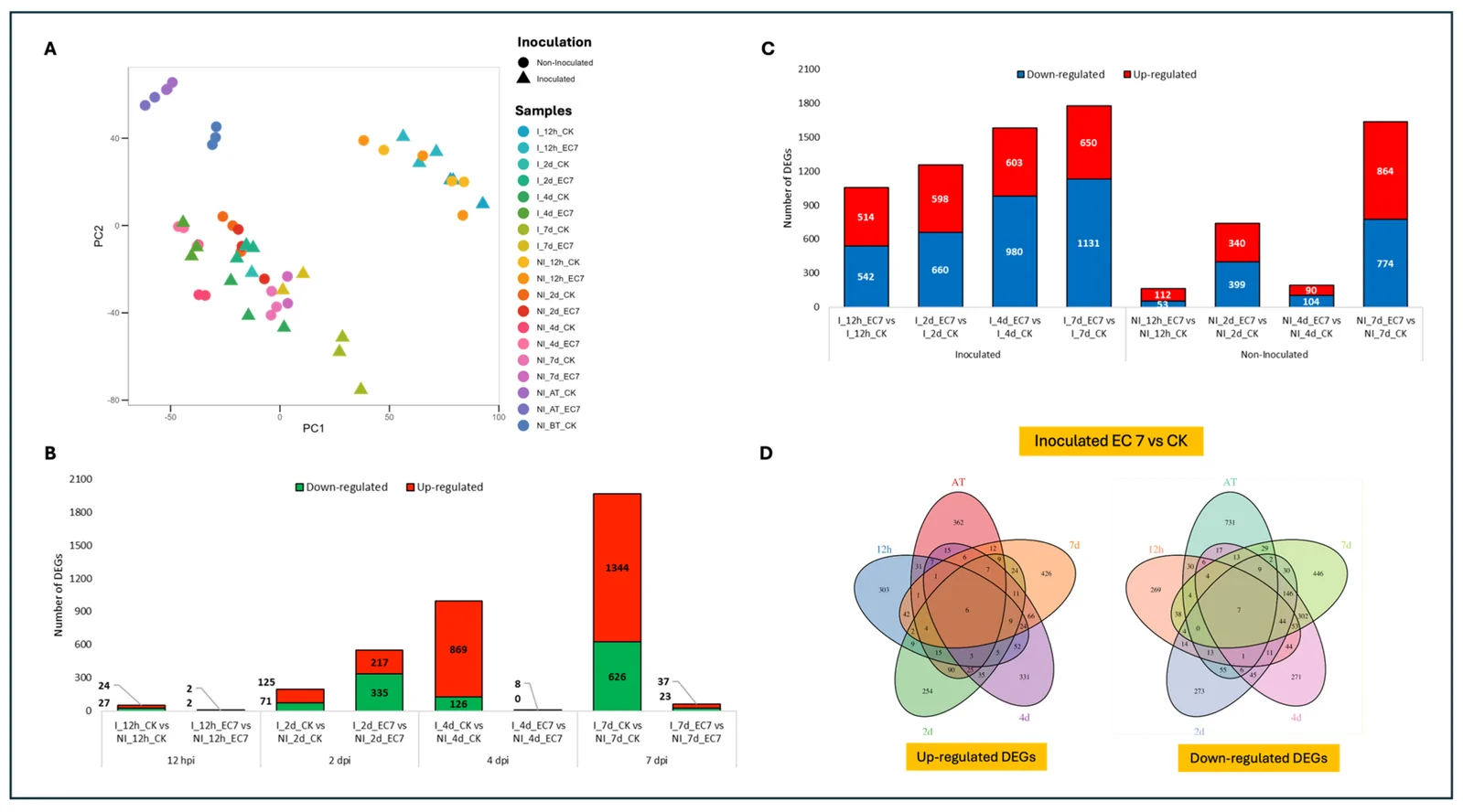
Transcriptomic comparison of inoculated (I) and non-inoculated (NI) tomato plants in EC 7 (electrical conductivity = 7 dS m^−1^) and the control (CK) treatments using DESeq2. CK across all sampled timepoints: before any treatment (BT), after initial salt treatments (AT), 12 h post-inoculation (12 hpi), 2, 4, and 7 days post-inoculation (dpi). (**A**) Principal component analysis (PCA) showing sample clustering based on gene expression profiles. (**B**) Number of differentially expressed genes (DEGs) between I vs. NI plants within each of CK and EC 7 treatments at 12 hpi, 2, 4, and 7 dpi. (**C**) Number of DEGs between EC 7 vs. CK treatments in each of I and NI plant groups over time. (**D**) Venn diagrams showing the number of upregulated and downregulated DEGs in inoculated EC 7 vs. CK at 12 hpi, 2, 4, and 7 dpi. Statistical significance was assessed using the Wald test, with adjusted *p*-value using the Benjamini–Hochberg method. Genes with adjusted *p*-value ≤ 0.05 and absolute log_2_ fold-change ≥ 1 were considered significant DEGs.

Samples were collected at multiple timepoints to capture the dynamics of transcriptional changes (Table S7, Figure 5). Principal component analysis (PCA) revealed that sample clustering was primarily driven by timepoint rather than by treatment or inoculation alone (Figure 5A). Three major clusters were observed: one comprising before treatment (BT) and after treatment (AT) samples, a second consisting of samples collected at 12 hpi, and a third containing the 2, 4, and 7 day post-inoculation (dpi) samples.

DEG analyses between inoculated (I) and non-inoculated (NI) plants was performed for each timepoint in each of CK and EC 7 treatments groups (I_12h_CK vs. NI_12h_CK, I_12h_EC 7 vs. NI_12h_EC 7, I_2d_CK vs. NI_2d_CK, I_2d_EC 7 vs. NI_2d_EC 7, I_4d_CK vs. NI_4d_CK, I_4d_EC 7 vs. NI_4d_EC 7, I_7d_CK vs. NI_7d_CK, and I_7d_EC 7 vs. NI_7d_EC 7) (Figure 5B). In CK plants, DESeq2 identified 51 (24 upregulated and 27 downregulated), 196 (125 up and 71 down), 995 (869 up and 126 down), and 1970 (1344 up and 626 down) DEGs at 12 hpi, 2 dpi, 4 dpi, and 7 dpi, respectively. In plants in the EC 7 treatment group, there were four (2 up and 2 down) at 12 hpi, 552 (217 up and 335 down) at 2 dpi, eight (8 up and 0 down) at 4 dpi, and 60 (37 up and 23 down) DEGs at 7 dpi, respectively. At all timepoints except 2 dpi, few DEGs were detected between inoculated and non-inoculated plants in the EC 7 treatment, suggesting that salt stress may mask the transcriptional response to *X. perforans* infection.

To further explore the effect of salt stress, comparisons between EC 7-treated plants and CK plants were made to identify changes in gene expression in each of inoculated (I_12h_EC 7 vs. I_12h_CK, I_2d_EC 7 vs. I_2d_CK, I_4d_EC 7 vs. I_4d_CK, and I_7d_EC 7 vs. I_7d_CK) and non-inoculated (NI_12h_EC 7 vs. NI_12h_CK, NI_2d_EC 7 vs. NI_2d_CK, NI_4d_EC 7 vs. NI_4d_CK, and NI_7d_EC 7 vs. NI_7d_CK) groups (Figure 5C). In inoculated plants, DESeq2 identified 1056 (514 upregulated and 542 downregulated), 1258 (598 up and 660 down), 1583 (603 up and 980 down), and 1781 (650 up and 1131 down) DEGs between EC 7 and CK treatments at 12 hpi, 2, 4, and 7 dpi, respectively. In non-inoculated plants, 165 (112 up and 53 down), 739 (340 up and 399 down), 194 (90 up and 104 down), and 1638 (864 up and 774 down) DEGs were detected at the same timepoints. These results indicate that while salt stress alone influences gene expression, most notably at 7 dpi, the presence of both salt stress and pathogen infection leads to a consistently higher number of DEGs across all timepoints. This suggests that simultaneous abiotic and biotic stress induce a broader transcriptional response in tomato.

Given the focus on understanding how salinity treatment alters the transcriptional response to *X. perforans* infection, further analyses centered on comparisons between inoculated plants in the EC 7 and CK treatments. A Venn diagram was used to visualize the overlap and uniqueness of DEGs across all timepoints in this comparison (Figure 5D). Among the upregulated DEGs, six genes were shared across all timepoints, while the number of unique upregulated genes at each timepoint was 362 at AT, 303 at 12 hpi, 254 at 2 dpi, 331 at 4 dpi, and 426 at 7 dpi. For downregulated DEGs, seven genes were common across all timepoints, with 731, 269, 273, 271, and 446 genes uniquely downregulated at AT, 12 hpi, 2 dpi, 4 dpi, and 7 dpi, respectively.

#### 2.6.3. GO Enrichment

**Figure 6:**
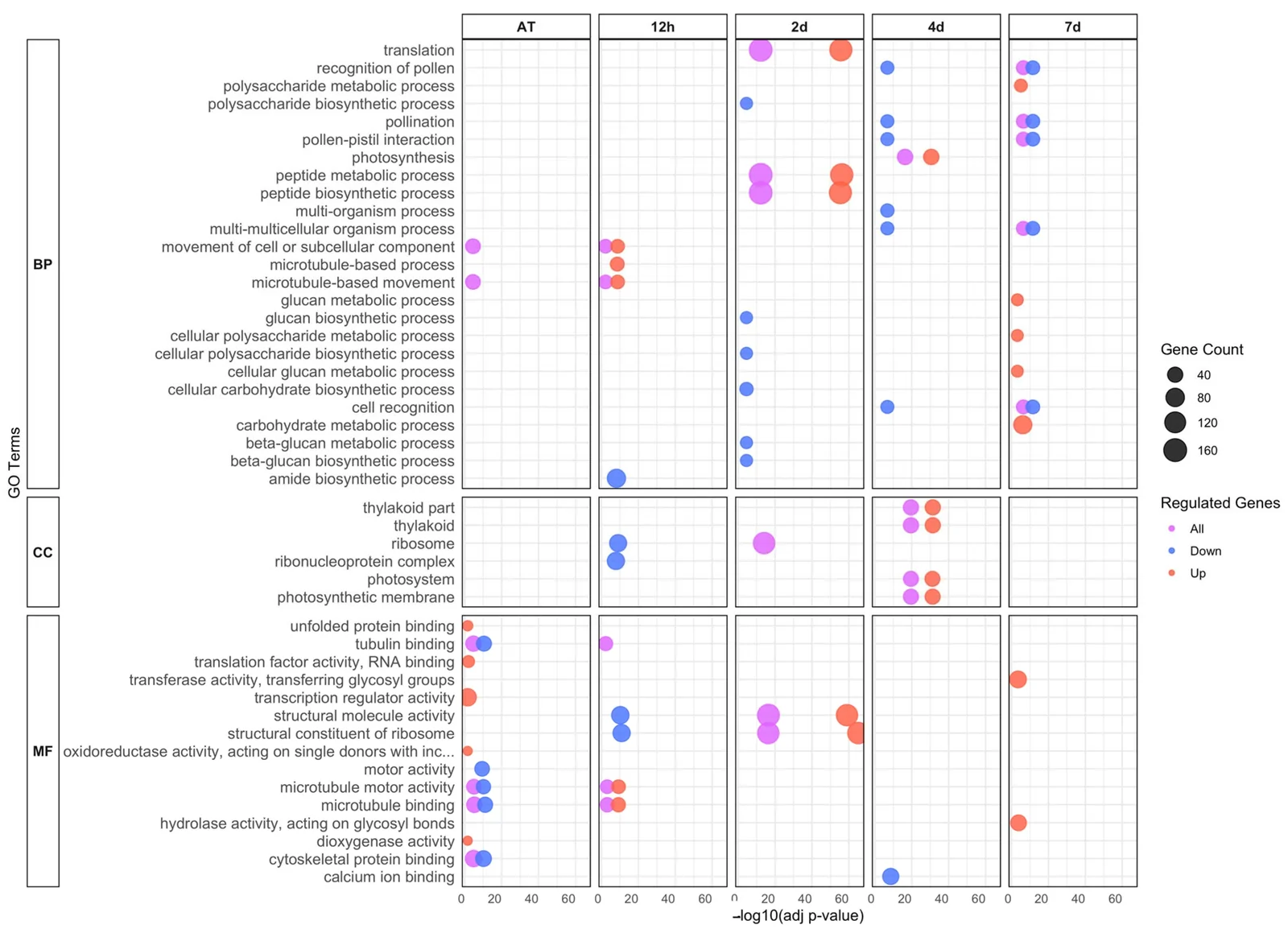
GO enrichment analysis of differentially expressed genes (DEGs) over time in EC 7 (electrical conductivity = 7 dS m^−1^) vs. CK (control) treatments. The bubble plot displays the top significantly enriched Gene Ontology (GO) terms at each timepoint (AT, 12 hpi, 2, 4, 7 dpi) across three domains: Biological Process (BP), Cellular Component (CC), and Molecular Function (MF). GO terms were identified from upregulated, downregulated, and all DEGs (based on DESeq2), with criteria: adj *p* value ≤ 0.05 and gene count ≥ 10. Bubble size indicates the number of genes enriched in each GO term and bubble color represents category of regulated genes.

GO enrichment analysis revealed distinct patterns of functional enrichment based on time post-inoculation (Figure 6, Table S8). In the early timepoints, AT and 12 hpi, most enriched genes were downregulated, particularly those involved in microtubule-based processes, motor activity, and intracellular transport (AT). Key processes related to translation and protein synthesis were also downregulated, indicating a reduction in protein production (12 hpi). However, by 12 hpi, genes linked to cytoskeletal remodeling and motor activity were upregulated, marking the onset of cellular reorganization.

A more significant increase was found at 2 to 4 dpi in gene counts and in upregulated genes associated with photosynthesis, ribosomal function, and protein synthesis. Significant enrichment of thylakoid, photosystem II oxygen evolving complex, and oxidoreductase activity reflects a metabolic shift toward enhanced energy production and biosynthesis. Meanwhile, downregulated genes were enriched in categories related to carbohydrate biosynthesis, vesicle-mediated transport, and ion channel activity, implying a reprioritization away from growth and signaling pathways to focus on metabolic restoration and stress acclimation.

By 7 dpi, gene expression indicated a transition toward recovery and structural reinforcement. GO terms related to cell wall organization, carbohydrate metabolism, and extracellular regional components (such as apoplast and glycosyltransferase activity) were significantly upregulated, suggesting enhanced cell wall remodeling and sugar metabolism critical for long-term stress tolerance. Simultaneously, genes involved in transcription regulation, oxidative stress response, and mitochondrial functions were downregulated, indicating a downshift in active defense and signaling pathways as the system stabilizes.

#### 2.6.4. KEGG Enrichment: Inoculated EC 7-Treated Plants vs. Inoculated CK Plants

**Figure 7:**
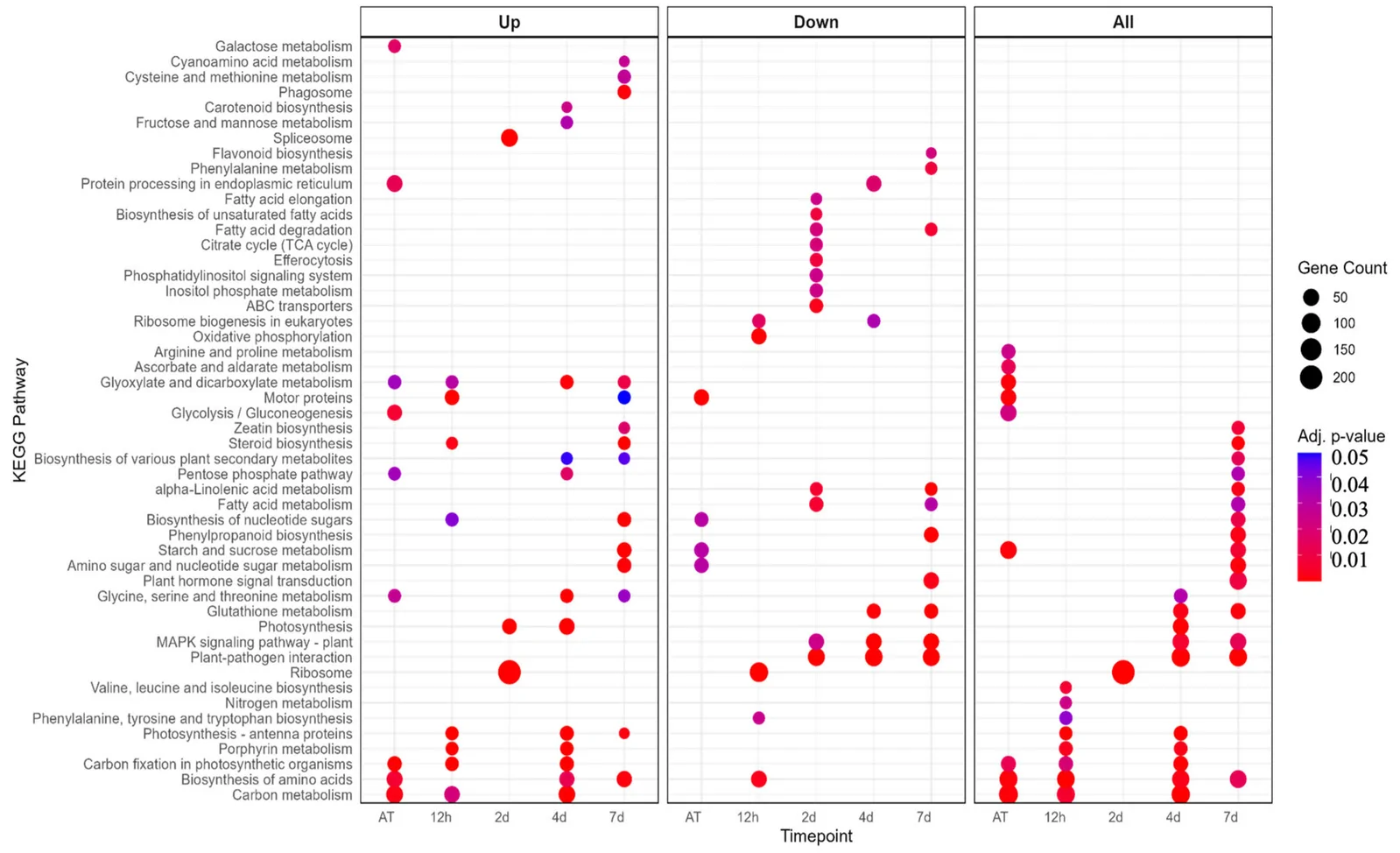
KEGG pathway enrichment analysis of differentially expressed genes (DEGs) across timepoints in EC 7 (electrical conductivity = 7 dS m^−1^) vs. CK (control) treatments. Bubble plots show the top significantly enriched Kyoto Encyclopedia of Genes and Genomes (KEGG) pathways at each timepoint (AT, 12 hpi, 2, 4, 7 dpi) for up- and downregulated, and all differentially expressed genes (DEGs). Only pathways with an adj *p*-value ≤ 0.05 and gene count ≥ 10 were included. Bubble size indicates the number of genes enriched in a pathway and bubble color represents the adj *p*-value.

Several pathways were significantly enriched in inoculated plants in the EC 7 treatment compared to inoculated plants in the CK treatment across multiple timepoints (AT, 12 hpi, 2, 4, 7 dpi) (Figure 7, Table S9). They fell into four major functional categories: energy and primary metabolism, photosynthesis-related processes, defense and signaling, and hormone and secondary metabolism. Among the most consistently enriched were carbon metabolism, amino acid biosynthesis, and carbon fixation in photosynthetic organisms, followed by MAPK signaling, plant–pathogen interaction (PPI), and glyoxylate and dicarboxylate metabolism. The most significantly enriched single pathway was the ribosome pathway at 2 days post-inoculation (2 dpi; adj *p* = 2.06 × 10^−91^, 208 genes), indicating a robust increase in protein synthesis in response to the salt and disease stress.

Tomato plants after the five initial salt treatments (AT) showed upregulation of carbon metabolism, carbon fixation, and amino acid biosynthesis, accompanied by downregulation of motor proteins, indicating a shift in energy allocation toward photosynthetic activity and the reduction in intracellular transport. By 12 hpi, motor proteins and photosynthesis-related pathways such as carbon fixation and porphyrin metabolism were upregulated, indicating increased investment in light-driven energy production. Meanwhile, suppression of ribosome biogenesis and oxidative phosphorylation pathways suggests a temporary reallocation of energy away from protein synthesis and mitochondrial respiration toward photosynthetic activity. From 12 hpi to 7 dpi, there was a sustained enrichment of one or more photosynthetic pathways.

At 2 dpi, ribosomal activity was strongly upregulated, along with spliceosome and sustained photosynthetic activity, whereas defense-associated pathways such as MAPK signaling, PPI, and fatty acid metabolism were downregulated, indicating a shift away from active defense toward recovery and metabolic rebuilding. This trend continued at 4 dpi, with enrichment in photosynthesis, porphyrin metabolism, and biosynthesis of secondary metabolites, alongside suppression of endoplasmic reticulum protein processing, glutathione metabolism, and stress signaling.

By 7 dpi, plants exhibited a distinct shift toward growth and recovery, marked by upregulation of starch and sucrose metabolism, amino sugar metabolism, and hormone biosynthesis pathways (zeatin, steroids, cysteine and methionine), as well as secondary metabolite pathways such as flavonoid and phenylpropanoid biosynthesis. Simultaneously, defense and signaling pathways, including MAPK signaling, hormone signaling, and glutathione metabolism, were broadly downregulated. Together, these patterns reflect a coordinated stress adaptation strategy involving early metabolic reprogramming, suppression of defense, and late-stage hormonal remodeling to support growth and repair under combined abiotic and biotic stress.

## 3. Discussion

Pre-exposing plants to a stressor, such as moderate to high salinity, prior to inoculation with a pathogen, can prime plant defense mechanisms and increase tolerance to subsequent stressors [28,29,30]. This pre-exposure can enhance germination uniformity, plant growth, and tolerance to a subsequent stress by establishing a “stress memory” that accelerates defense activation [18,25,27,31]. For instance, treating tomato seeds with 300 mM NaCl (30 dS m^−1^) for 24 h improved germination, seedling vigor, and tolerance to both salinity and pathogens such as *Ralstonia solanacearum*, the causal agent of bacterial wilt in tomatoes [25]. Consistent with these findings, salinity treatments in our study strengthened defense against *X. perforans* and reduced BST severity compared to untreated controls. However, these benefits came with notable physiological trade-offs.

Tomatoes are considered moderately tolerant to salt stress [4]. However, high salinity can result in reduced seed germination, plant growth, biomass, and final fruit yield [32,33,34]. In our greenhouse trials, salinity treatments were defined by the EC of the irrigation solutions (EC 0.5 [control], EC 3, EC 5, and EC 7) rather than by a target EC level in the leachate or growing medium. Repeated application of salt solutions was intended to generate progressive salt accumulation in the soil, with higher EC treatments resulting in greater salt accumulation over time. This approach reflects agricultural conditions where repeated exposure of saline irrigation water and fertilizer application can lead to progressive salt accumulation in the root zone. This accumulation resulted in reduced plant height and fresh shoot weight compared to controls. These effects were more pronounced at EC 5 and EC 7, indicating that physiological stress intensified with repeated exposure at higher salinity levels. In contrast, EC 3 produced a more limited and inconsistent response, significantly affecting only non-inoculated plant weight without broader impacts on other measured parameters. Fresh shoot weight was significantly affected by the interaction between salt treatment and inoculation. Despite reduced disease severity, inoculated plants generally had lower fresh shoot weight than non-inoculated plants, suggesting a potential physiological cost associated with stress adaptation. Together, these results suggest that increasing salinity negatively impacted plant growth, likely through physiological constraints associated with salt stress.

Salinity treatments significantly altered key leaf gas exchange variables, with decreases in net CO_2_ assimilation (*A*), transpiration (*E*), and stomatal conductance (*g_s_*) across all salt-treated tomato plants compared to the controls. These reductions indicate that osmotic stress induced by the salinity treatments was the dominant physiological stressor. This stress reduced stomatal conductance and transpiration and may have limited pathogen ingress, thereby overshadowing any measurable effects of bacterial infection [35]. Studies have shown that increasing salinity can increase the osmotic pressure in soil, thereby lowering water availability and limiting water uptake by the roots [22]. This mechanism was reflected in our greenhouse trials, where salt-treated soils retained moisture longer than controls. In turn, plants respond by restricting leaf expansion and closing stomata to maintain osmotic balance, though this comes at the cost of limited photosynthesis due to reduced CO_2_ uptake through the stomata [4,35]. These physiological adjustments manifest as chlorosis, wilting, and stunted growth, all of which are characteristic of systemic salt stress [32,33,34].

In the present study, salt-induced stomatal closure potentially served a dual protective role: mitigating water loss and limiting bacterial invasion. Given that bacteria enter plant tissues through natural openings such as stomata or through wounds [36], reduced stomatal aperture under salt stress can limit pathogen entry [35]. Consistent with this, inoculation with *X. perforans* had little impact on leaf gas exchange, as similar values were measured in both inoculated and non-inoculated plants. Similarly, in vitro and in planta population assays showed no differences between control and salt-treated plants, with bacterial populations continuing to increase despite salt treatments. The in vitro population assay was only performed to determine whether salinity directly affected bacterial growth, whereas the in planta population assay used infiltration to bypass bacterial entry and initial colonization, allowing the evaluation of whether salinity could directly affect bacterial multiplication within leaf tissues. These findings indicate that NaCl at tested levels did not directly inhibit *X. perforans* growth. Instead, the observed reduction in BST severity is best explained by salt-induced changes in host physiology that restricts infection, rather than by direct effects on the pathogen [31,37]. Therefore, although infiltration is commonly used for transcriptomic studies, tomato plants were inoculated by foliar spray to preserve the natural infection process and capture host transcriptional responses to salinity during *X. perforans* infection. In contrast, direct salt-mediated changes in plant–pathogen interactions have been reported in other systems. For example, in *Arabidopsis* exposed to 300 mM NaCl (30 dS m^−1^), disease severity was reduced, as indicated by smaller lesions caused by *Pseudomonas syringae* pv. *maculicola* DG3 [37]. In that system, disease reduction was attributed to ion accumulation in the host’s intracellular spaces, which limited bacterial colonization [37,38]. Future studies using microscopy to directly visualize stomatal aperture during salinity and *X. perforans* infection would help validate whether salt-induced stomatal closure contributes to reduced pathogen entry. Additionally, quantifying salt accumulation in the apoplast could clarify the potential role of intercellular ion accumulation in mediating disease suppression.

KEGG enrichment revealed significant downregulation of the MAPK signaling and PPI pathways, both of which are central to pattern-triggered immunity (PTI). PTI is initiated by pattern recognition receptors such as FLS2, which perceives bacterial flagellin and activates downstream signaling through co-receptors, MAPK cascades, WRKY transcription factors, and ROS production [39,40,41]. Although FLS2 itself was not differentially expressed in our dataset, multiple downstream PTI components, including MPK3, MKK2/MKK4, WRKY33, PR1 isoforms, SERK3A/B, EDS1, CNGC15, and RBOH1, were significantly suppressed under salt stress. Therefore, active pathogen-induced defense was not triggered. This coordinated repression reflects a reprioritization of resources from bacterial defense. A limitation of this study is that transcriptome sampling did not include early timepoints within the first 0–6 hpi. Although samples were collected at 12 hpi and later stages of disease development, transient early transcriptional responses associated with pathogen recognition and initial defense activation may not have been captured.

In EC 7-treated plants, very few DEGs were detected between inoculated and non-inoculated samples at all timepoints, apart from 2 dpi, indicating a minimal transcriptional response to bacterial inoculation. This suggests that the high salinity induced a strong response in tomato, causing both inoculated and non-inoculated plants to show similar transcriptional patterns, explaining the limited DEGs. Another potential explanation for the low number of DEGs could be the limited disease development observed in EC 7-treated plants. Therefore, because disease severity was minimal, the associated transcriptional response was also low, resulting in fewer detectable changes in gene expression. In contrast, control plants exhibited a much stronger transcriptional response to infection, particularly at 7 dpi, consistent with the more pronounced disease symptoms observed in these plants. Together, these results indicate that BST primarily drives transcriptional changes under non-saline conditions, whereas salt stress masks infection-associated transcriptional responses. Another reason could be the overlap in transcriptional pathways involved in salt stress and disease response. A recent comparative transcriptomic study in tomato identified 1474 DEGs commonly shared between biotic and abiotic stresses, highlighting key regulatory pathways, including hormone signaling, transcription factors, calcium signaling, and cell wall metabolism [42]. Salinity stress is known to trigger extensive transcriptional reprogramming in tomato, affecting pathways related to ion homeostasis, detoxification, metabolism, and oxidative stress [4,22]. This overlap suggests potential competition for regulatory resources between stresses, allowing dominant factors like salinity to reprogram the transcriptional response to prioritize abiotic stress tolerance [42].

In place of active immune signaling, salinity treatments induce comprehensive ROS management through metabolic and cellular reprogramming that supports survival under stress [18,22,25,37,38]. Salinity imposes both osmotic and ionic stress, disrupting cellular homeostasis and altering hormone signaling, particularly abscisic acid (ABA) and indole-3-acetic acid (IAA), which regulate processes such as stomatal closure to reduce water loss and adjust growth patterns, helping the plant adapt to the stress [4,32]. At 4–7 dpi, genes involved in MAPK signaling and plant hormone signal transduction, including *RBOH1*, *SlIAA16*, and *SlLAX1*, were upregulated. These genes are associated with ROS-mediated ABA/stomatal regulation and auxin signaling [43,44,45], suggesting that modulation of hormone responses and stomatal control contributes to tomato adaptation under stress conditions. The major consequence of elevated ROS levels is oxidative damage to plant cells. To counteract this, plants activate antioxidant enzymes such as superoxide dismutase (SOD), catalase (CAT), and glutathione peroxidase (GPx), which neutralize harmful oxidative molecules produced under salt stress [4,22,46].

KEGG enrichment revealed strong upregulation of pathways associated with carbon metabolism, including carbon fixation and carbohydrate metabolism across timepoints. This coordinated response is further supported by the expression of central metabolic genes, such as *SlFBA1/2/6/7*, *rbcS1*, *GPI*, *MDH*, and *CAT2*, which integrates carbon flux with ROS detoxification processes. Early upregulation of photosynthesis-related genes, including light-harvesting *chlorophyll a/b-binding (CAB)* proteins and the photoprotective factor *psbS*, suggests enhanced dissipation of excess excitation energy, thereby limiting ROS generation in the chloroplast and contributing to photoprotection under conditions where photosynthetic carbon assimilation is constrained [35,47]. Concurrent enrichment of porphyrin metabolism, including *POR2* and *GSAAT*, point to regulation of chlorophyll biosynthesis, likely minimizing the accumulation of phototoxic intermediates that could exacerbate ROS production [48,49,50].

However, the upregulation of photosynthesis and carbon metabolism-related pathways does not necessarily indicate enhanced photosynthetic performance, as physiological measurements showed significant reductions in *A*, *E*, and *g_s_* under salinity treatments. Instead, these transcriptional changes suggest an acclimation response aimed at maintaining photosynthetic machinery, regulating carbon flux, and mitigating oxidative stress. Although genes involved in photoprotection, carbon fixation, and ROS detoxification were upregulated, these molecular adjustments were insufficient to overcome salinity-induced physiological limitations, including reduced stomatal conductance and CO_2_ diffusion, resulting in an overall decline in leaf gas exchange.

Consistent with these adjustments, citrate cycle-associated (TCA) enzymes such as *ICDH1* and *Slα-KGDH* were also enriched, indicating increased respiratory activity to generate ATP and metabolic intermediates required for stress tolerance [51]. Although the upregulation of photosynthesis and light-harvesting components may help elevated energy demands under salt stress, it also increases susceptibility to light-induced damage. Excess excitation above tolerance in chloroplasts can generate ROS, leading to photoinhibition and oxidative damage [52]. This imbalance was partially mitigated by 4 dpi through upregulation of carotenoid biosynthesis genes such as *PSY1* and *VDE*, which facilitate the dissipation of excess light energy and reduce oxidative damage [53]. In parallel, enrichment of the pentose phosphate pathway suggests an additional layer of redox regulation, as this pathway generates NADPH required to sustain antioxidant systems, including glutathione and ascorbate recycling [54].

At later stages (4–7 dpi), the transcriptional profile shifts from early stress mitigation, such as protection of the photosynthetic apparatus, toward longer-term adaptation under salt stress. Enrichment of amino acid biosynthesis, carbohydrate metabolism, and secondary metabolite pathways indicates sustained metabolic reprogramming. Activation of hormone-related pathways, including zeatin and steroid biosynthesis, along with upregulation of *SAM3*, *CKX1*, and *CAS1*, highlights an increasing role for hormonal signaling in coordinating growth and stress tolerance [55,56,57]. Moreover, the enrichment of endoplasmic reticulum protein-processing pathways, particularly heat shock proteins such as *HSP70* and *HSP90*, shows that maintaining protein homeostasis is a critical component of prolonged stress tolerance [58].

Additionally, KEGG analysis revealed an enrichment of arginine and proline metabolism, starch and sucrose metabolism, amino acid biosynthesis, and antioxidant pathways associated with osmotic stress adaption and osmoprotectants. Osmoprotectants such as proline and soluble sugars help maintain osmotic balance and protect against dehydration in the EC 7-treated tomato plants [4,59]. The upregulation of sugar metabolism genes, including *GolS2*, together with the enrichment of pathways involved in osmoprotectant production, suggest a shift toward osmotic adjustment and redox buffering [60]. These compatible solutes likely play dual roles by stabilizing cellular structures and contributing to antioxidant capacity under sustained salinity stress.

These transcriptional changes are reflected at the physiological level, where salt-treated plants showed a slight increase in osmolality and fruits that were perceived as sweeter by taste-panel participants, particularly in EC 5 and EC 7 treatments. Although changes in solute concentration and osmotic adjustment were not statistically significant within the scope of this experiment, the observed trend suggests a potential contribution of osmotic regulation to the plant response under salt stress. The enhanced sweetness may be associated with multiple physiological adjustments, including reduced leaf and fruit size, solute concentration effects, and enhanced carbohydrate metabolism, such as increased starch accumulation during early fruit development followed by its conversion to sugars during ripening [59]. Furthermore, osmolality experiments revealed that salt-treated plants had higher solute concentrations and showed evidence of osmotic adjustment, which is a key mechanism in plants to cope with salinity stress by accumulating solutes to maintain cellular water balance [4,59,61]. Collectively, the osmolality measurements, sensory evaluation, and transcriptomic analyses indicate higher solute concentrations and altered sugar metabolism under salinity stress, suggesting that salinity may influence tomato fruit sugar composition. However, direct quantification of soluble sugars using °Brix or HPLC is required to confirm whether sugar accumulation increases under salinity.

A higher incidence of blossom-end rot was observed in salt-treated fruit compared to the control. This may result from high salinity interfering with calcium transport to the fruit or gradual nutrient depletion in the soil. Calcium is transported via the xylem, and reduced transpiration has been shown to limit calcium delivery to developing tissues, leading to disorders such as blossom-end rot [62]. Consistent with this mechanism, high salinity in our study decreased transpiration, likely restricting calcium transport and contributing to the increased incidence of blossom-end rot [63]. KEGG analysis further revealed downregulation of calcium-associated signaling components, including *CNGC* channels, calmodulin (*SlCaM6*), and *CDPK1*, along with suppression of MAPK and phosphatidylinositol signaling pathways, indicating a reduced capacity for calcium uptake and downstream calcium signal transduction under sustained salt stress [64,65,66,67]. Future studies should investigate whether optimizing nutrient availability, such as calcium, can reduce blossom-end rot while maintaining the flavor benefits of the higher salinity treatments. Collectively, these results indicate that inoculated EC 7-treated tomato plants enhance salt tolerance through a multi-layered strategy that limits ROS production, reinforces antioxidant defenses, and preserves cellular integrity, thereby maintaining redox homeostasis. Also, reinforcing the idea that energy and oxidative stress management are prioritized over pathogen defense.

Growers require solutions to effectively address the impact of salinization on crops and disease, and explore the potential strategies for its management. The findings of the study are based on a single tomato cultivar (FL 47), bacterial strain (QL), NaCl-induced salt stress, and greenhouse conditions. Further validation across diverse tomato genotypes, pathogen strains, salt-induced salinity stress, and production environments is needed to assess their broader applicability. Future research should be conducted to determine the range of soil salinity levels for reducing BST without compromising fruit yield and to explore the effects of salinity on BST under field conditions, with particular attention to its impacts on disease pressure, yield, and fruit quality. Additionally, breeding tomato cultivars with enhanced tolerance to both salinity and disease could offer a strategic approach to crop improvement. Understanding the role of stomatal aperture in disease suppression may also provide deeper insight into plant responses under combined stress conditions. A limitation of this study is that the transcriptomic analysis focused on the inoculated EC 7 and control comparison and therefore did not fully evaluate the separate and combined effects of salinity and pathogen infection using a full factorial model. Additionally, independent validation of key DEGs was not performed. Future studies should incorporate qRT-PCR analysis of selected key DEGs (such as MPK3, WRKY33, PR1, rbcS1, and GolS2) to further confirm and validate transcriptomic patterns identified through RNA-seq analysis. While salinity is often viewed as a limiting factor in agriculture, our findings suggest that low to moderate salinity may achieve a better balance between disease reduction and yield preservation in controlled environments.

## 4. Materials and Methods

### 4.1. Preparation for Salinity Treatments

Salinity treatments were based on electrical conductivity (EC), which reflects the concentration of dissolved salts by measuring a solution’s ability to conduct electricity. Salinity treatments were prepared by dissolving NaCl (≥99.0%; Thermo Fisher Scientific, Waltham, MA, USA) in tap water (basal EC of approx. 0.5 dS m^−1^). In this study, the treatments were defined by the EC of the irrigation water rather than a target soil EC: EC 0.5 dS m^−1^ (control), EC 3 dS m^−1^ (1.45 g NaCl/L), EC 5 dS m^−1^ (2.59 g NaCl/L), and EC 7 dS m^−1^ (3.50 g NaCl/L). The different EC levels were designed to produce different rates of salt accumulation over time. Final EC measurements were confirmed using a benchtop EC meter (Fisherbrand accument AR60; Thermo Fisher Scientific, Waltham, MA, USA).

### 4.2. Preparation of X. perforans Bacterial Suspension

The *X. perforans* strain QL, which was isolated from diseased tomato plants in Homestead, FL, USA, was used for all inoculations in this study. Bacterial cells were recovered from −80 °C storage by streaking onto nutrient agar (NA; Thermo Fisher Scientific, Waltham, MA, USA) and plates were then incubated at 28 °C for 24 h. A loopful of bacterial cells was then re-streaked onto fresh NA plates, which were incubated for another 24 h. Bacterial cells were removed from the agar surface and suspended in sterile tap water (STW). The suspension was adjusted to ~5 × 10^8^ colony-forming units (CFU)/mL based on an optical density at 600 nm (OD_600_ = 0.3). For experiments where lower concentrations were used, the suspension was serially diluted in STW.

### 4.3. Impact of Salinity on Leaf Gas Exchange, Plant Growth, and BST

To evaluate the effects of salinity on tomato plant growth and BST, the experiment included two groups: inoculated and non-inoculated tomato plants. Inoculated plants were used to assess the impact of salinity on BST development, while non-inoculated plants served as controls to characterize the effects of salinity stress alone. Four-week-old tomato seedlings (cv. FL 47) were transplanted into 15 cm pots filled with a commercial potting mix (Pro-Line C/B HydraFiber blend, Jolly Gardener, Poland Spring, ME, USA). After transplanting, plants were allowed to acclimate for one week before the salt treatments began. Plants were manually irrigated via soil drench every two days with 150 mL of salt solutions (EC 3, EC 5, or EC 7 dS m^−1^). The purpose of the repeated applications of salt treatments was intended to generate progressive salt accumulation in the soil, with higher EC treatments producing greater and more rapid salt buildup over time, while lower EC treatments resulted in slower accumulation. Tap water with no salt added (EC ~0.5 dS m^−1^) served as the untreated control. Styrofoam plates were placed under the pots to capture any leachate.

After irrigating plants five times with salt solutions (8 days after treatments started), half of the plants were inoculated with a suspension of *X. perforans* at ~5 × 10^8^ CFU/mL and the inoculated plants were placed in a moist chamber for 48 h for disease promotion. Non-inoculated plants were sprayed with STW and placed in a separate moist chamber for consistency and to prevent contamination by the pathogen. After 48 h, all plants were arranged on the bench in a randomized complete block design, and irrigation treatments were applied directly to the soil. Irrigation of treatments continued for the duration of the experiments. Following the onset of BST symptoms (within 5–7 days post inoculation), disease severity was assessed by visually estimating the percentage of diseased area on three leaves randomly selected between the third and fifth nodes below the apical meristem of each plant. The area under the disease progress curve (AUDPC) was calculated based on disease severity ratings collected over the experimental period. Plant height was measured from the soil surface to the shoot apex, and shoot fresh weight was recorded after cutting the stem at the soil surface. Soil and leachate (drained from the bottom of each pot) samples were also collected to determine the accumulated salinity.

To assess physiological plant response to salt treatments and BST, leaf gas exchange [net CO_2_ assimilation (*A*), transpiration (*E*), and stomatal conductance (*g_s_*)] was measured with a CIRAS-3 portable gas exchange analyzer (PP Systems, Amesbury, MA, USA). Measurements were made biweekly on one fully expanded leaflet from the upper-middle section of the canopy. During leaf gas exchange measurements, the following parameters were set: air flow rate into the leaf cuvette at 300 mL min^−1^, photosynthetic photon flux inside the cuvette at 1000 μmol quanta m^−2^ s^−1^, the reference CO_2_ concentration in the leaf cuvette at 390 μmol CO_2_ mol^−1^, the air temperature in the cuvette at 25 °C, and the relative humidity at 70%. There were five replicates per treatment with each single plant as a replicate. The experiment was conducted twice.

### 4.4. Electrical Conductivity of Water Leachate and Soil Samples as Affected by Salinity Treatment

Quantification of soil salinity was conducted with leachate and soil samples collected from pots of the control and salt-treated plants (EC 3, 5, and 7 dS m^−1^). Water leachate draining from the holes in the bottom of each pot was collected at the beginning and end of the experiment and filtered through medium-flow-rate filter paper (Thermo Fisher Scientific, Waltham, MA, USA). Soil samples were collected, placed in paper bags, and oven-dried at 40 °C for approximately 48 h. Dried soil samples were screened through a 2 mm sieve, and 2 g of soil was mixed with 20 mL of double-distilled (dd) water. The resulting soil suspension was filtered using medium-flow-rate filter paper, and the filtrate was collected. Filtrates for water leachate and soil samples were measured with a benchtop EC meter (Fisherbrand accument AR60; Thermo Fisher Scientific, Waltham, MA, USA). There were three replicates per treatment, and the experiment was conducted twice.

### 4.5. Impact of Salt Treatments on X. perforans Bacterial Population

#### 4.5.1. In Vitro Activity of Salinity Treatments Against Bacterial Populations

To determine whether salinity could directly affect *X. perforans* (QL) growth, an in vitro assay was conducted using salinity treatments (EC 3, 5, or 7 dS m^−1^) prepared using NaCl and STW as previously described (adjusted for smaller volume). A bacterial suspension was prepared as previously stated and adjusted to 10^5^ CFU/mL. Then, 20 μL of the suspension was added to tubes containing 2 mL of saline solution. Additionally, three tubes containing 2 mL of STW and 20 μL of the bacterial suspension served as the control. All tubes were incubated at 28 °C on an orbital shaker at 150 rpm for 0, 1, 4, and 24 h. At each timepoint, 50 μL of the suspension from each tube was streaked onto each NA plate, and the plates were incubated at 28 °C for 48 h. Colonies were counted, and the CFU/mL was calculated. Each treatment consisted of three replicates, and the experiment was conducted two times.

#### 4.5.2. In Planta Bacterial Populations in Leaf Apoplast

To determine whether salinity could directly affect *X. perforans* (QL) populations in planta, a leaf infiltration assay was conducted. Four-week-old tomato plants were irrigated with 150 mL of salt solutions (EC of 3, 5, or 7 dS m^−1^) and tap water (control) via soil drench every two days. After the fifth application, three leaflets per plant, located between the third and fifth nodes below the apical meristem, were infiltrated using a 3 mL syringe with a suspension of *X. perforans* (strain QL) at 10^5^ CFU/mL, prepared in sterile double-deionized (DD) water amended with 0.01 M MgSO_4_. The inoculated area was marked by small perforations along the margin for identification after drying. Following inoculation, plants were allowed to air-dry for 1 h. At each timepoint (0, 3, 6, and 9 days post-inoculation), an infiltrated leaflet was excised, and two leaf disks (0.5 cm^2^) were collected from the inoculated region. The leaf disks were homogenized in 1 mL of 0.01 M MgSO_4_ solution using a sterile glass rod in a test tube. Serial 1:10 dilutions were prepared by adding 50 µL of homogenate to 450 µL of 0.01 M MgSO_4_, making dilutions up to 10^3^ for day 0 samples and up to 10^7^ for days 3, 6, and 9. Aliquots (50 µL) from each dilution were streaked onto each NA plate and the plates were incubated at 28 °C for 48 h. The CFU/cm^2^ was calculated based on the following equation:

(1) $$CFU\;per\;{\mathrm{cm}}^{2}\;=\frac{C\times D\times Vtotal\;}{Vplated},$$

where C = number of colonies counted, D = dilution factor, Vtotal = total volume to extrapolate (typically 1000 µL = 1 mL), and Vplated = Volume plated (in µL). Each plant consisted of three biological replicates, and the experiment was conducted twice.

### 4.6. Osmotic Adjustment of Plants in Response to Salinity

Four-week-old tomato plants (cv. FL 47) were irrigated via soil drench with 150 mL of tap water (control) and salt solutions (EC 3, 5, and 7 dS m^−1^) every two days for four weeks. At the end of the treatment period, three leaflets located between the third and fifth nodes below the apical meristem were collected and submerged in DD water at 4 °C in the dark for 24 h for full turgidity. Then leaflets were drained, patted dry, and homogenized in a mortar using pestle to extract sap. The sap was then centrifuged at 10,000× *g* for 20 min at 4 °C. The resulting supernatant was transferred to 2 mL tubes and stored at 4 °C until further use. Then 10 µL of each sap sample was placed on a filter paper disk and loaded into a vapor pressure osmometer (Vapro5600, Wescor, Inc., Logan, UT, USA) to measure leaf sap osmolality, the concentration of osmotically active dissolved particles (e.g., sugars and ions such as potassium) in sap extracted from leaflets. Osmotic potential at full turgor (Ψs100) was calculated using the van’t Hoff equation [68]:

(2) Ψs100= −(C × R ×T),

where C = osmolality (mmol/kg), R = universal gas constant (0.00831 MPa·kg·mol^−1^·K^−1^), and T = absolute temperature (298 K). Osmotic adjustment (OA), which is the process by which plants accumulate solutes to maintain water uptake and turgor under salt stress [69], was determined by subtracting the osmotic potential of stressed plants (Ψs100 stress) from that of control plants (Ψs100 control), using the formula:

(3) OA=Ψs100 control−Ψs100 stress 

The experiment included twelve replicates and was conducted two times.

### 4.7. Impact of Salinity on Fruit Quality and Flavor

To evaluate the effects of salinity on tomato fruit quality, a blind taste panel was formed with 32 participants. Tomato plants were grown in a greenhouse under the same conditions as previously described, except they were transplanted into 19 cm pots and irrigated every two days with 250 mL of tap water (control) and salt solutions (EC 3, 5, or 7 dS m^−1^) until harvest, about three months later. Each treatment had three replicates.

Tomato fruit were harvested at the 50–75% color break stage and ripened in paper bags at room temperature (20–21 °C) for 7–9 days. Total fruit yield was calculated using the pooled fruit from all replicates within each treatment. Once fully red, tomatoes were washed, cut, and presented to participants for a blind tasting. Each participant completed a standardized evaluation form (Figure S1) with rating attributes including appearance, sweetness, acidity, texture, and overall flavor. Treatment identities were disclosed after evaluations were submitted.

### 4.8. Transcriptomic Analyses

#### 4.8.1. Pathogen Inoculation and Sample Collection

To evaluate the changes in gene expression between salt-treated and control plants, an RNA-seq experiment was conducted in a temperature-controlled greenhouse in the Department of Plant Pathology at the University of Florida in Gainesville, FL. Treatments included EC 7 and control plants, both with and without inoculation. Tomato plants and treatments were prepared as previously described above in “Impact of salinity on leaf gas exchange, plant growth, and BST” with modifications for sample collection for RNA extraction. Each sample was collected from a different plant. A total of 57 samples were collected, with three biological replicates (one plant per replicate) for each experimental group at each sampling time (Table S7).

Before the start of any treatment, leaf tissue samples were collected from untreated plants (BT) and flash-frozen in liquid nitrogen as a baseline. Following BT collection, the remaining plants were subjected to the designated salt treatments. After the fifth salt treatment, leaf tissue was collected as the after-treatment (AT) sample and then flash-frozen. The remaining plants were then inoculated, and covered with clear polyethylene bags, which were secured around the pots with rubber bands, and maintained for 48 h to promote BST disease development. Non-inoculated plants were mock-treated with STW and bagged in the same manner. After 48 h, all plants were unbagged and arranged in a completely randomized block design. Leaf tissue samples were collected at 12 h post-inoculation (hpi), 2 days post-inoculation (dpi), 4 dpi, and 7 dpi and immediately flash-frozen. All collected tissue samples were homogenized using a Qiagen TissueLyser II (Qiagen GmbH, Hilden, Germany), and stored at −80 °C for RNA extraction. Disease severity was assessed by visually estimating the percentage of disease of the whole plant, and AUDPC was calculated based on disease severity ratings collected over the experimental period.

#### 4.8.2. RNA Extraction and Sequencing

Total RNA was extracted using the RNeasy Plant Mini Kit (Qiagen GmbH, Hilden, Germany), following the manufacturer’s protocol and stored at −20 °C. RNA quantity and purity were assessed with a NanoDrop spectrophotometer (Thermo Fisher Scientific, Waltham, MA, USA) and RNA samples were sent to Novogene Co. Inc. (Sacramento, CA, USA) for library preparation, sequencing, and data analysis. Libraries were constructed from messenger RNA (mRNA) purified from total RNA using poly-T oligo-attached magnetic beads. After fragmentation, first-strand cDNA was synthesized using random hexamer primers, followed by second-strand synthesis with either dTTP for non-strand-specific libraries or dUTP for strand-specific libraries. The libraries were quantified using Qubit (Life Technologies, Carlsbad, CA, USA) and real-time PCR (Applied Biosystems, Inc., Foster City, CA, USA), and their size distribution was assessed with a Bioanalyzer (Agilent Technologies, Santa Clara, CA, USA). Quantified libraries were pooled based on effective concentration and desired data yield, then sequenced usingIllumina NovaSeq X Plus (Illumina Inc, San Diego, CA, USA) to generate paired-end reads. Raw sequencing data were processed by adapter trimming and quality filtering. All data have been deposited in the NCBI Sequence Read Archive (SRA) under BioProject number PRJNA1303723.

#### 4.8.3. Transcriptomic Analysis Pipeline

The original image data file from high-throughput sequencing platforms (like Illumina) was transformed into sequenced reads (called Raw Data or Raw Reads) by CASAVA base recognition (Base Calling). Initial processing was done by Novogene’s bioinformatics team, followed by additional processing on the University of Florida’s HiPerGator supercomputing system. Quality of the sequences was assessed using FastQC (v0.12.0) [70]. Reads were then aligned to the tomato reference genome (*Solanum lycopersicum* cv. Heinz 1706, SL3.1; NCBI Accession GCF_000188115.5) using HISAT2 (v2.0.5) [71]. Transcript assembly was performed using StringTie (v1.3.3b) [72], and gene-level quantification was performed using featureCounts (v1.5.0-p3) [73].

#### 4.8.4. Differential Gene Expression Analysis

Differential expression analysis was performed using the DESeq2 (v1.40.2) [74] package in R (v4.4.2 ). Raw read counts obtained from featureCounts were used to identify differentially expressed genes (DEGs) across treatment conditions. Gene expression was compared over time between inoculated (I) and non-inoculated (NI) treatments (I EC 7 vs. NI EC 7; I CK vs. NI CK) to assess disease effects. Additionally, comparisons between salt treatments (I EC 7 vs. I CK; NI EC 7 vs. NI CK) were conducted to evaluate the impacts of salt stress on gene expression changes in the presence or absence of disease over sampled timepoints. Statistical significance was assessed using the Wald test, with *p*-values adjusted (adj *p*-value) using the Benjamini–Hochberg method [75]. Genes with adjusted *p*-value ≤ 0.05 and absolute log_2_ fold-change ≥ 1 were considered significant DEGs.

#### 4.8.5. Pathway Enrichment Analysis

Enrichment analysis focused on DEGs between inoculated salt-treated (I EC 7) and control (I CK) plants to specifically examine how salt stress modifies the transcriptional response to *X. perforans* infection (I EC 7 vs. I CK) across sampled timepoints. Analyses were conducted using the ClusterProfiler package (v4.18.4) [76] in R (v4.4.2). Gene Ontology (GO) and Kyoto Encyclopedia of Genes and Genomes (KEGG) enrichment analyses were conducted on DEGs, including upregulated, downregulated, and total subsets. Analyses were carried out across five timepoints (AT, 12 hpi, and 2, 4, and 7 dpi) to capture condition-specific and time-dependent responses. GO and KEGG annotations provided by Novogene were filtered to retain terms with adj *p*-values ≤ 0.05 and gene counts ≥ 10. The top enriched terms (based on gene count) were visualized using customized dots and bubble plots in ggplot2 (v4.0.3) [77], with dot size indicating gene count and color representing statistical significance.

### 4.9. Statistical Analyses

All statistical analyses were conducted using SAS (v9.4, SAS Institute Inc., Cary, NC, USA). For plant growth and leaf gas exchange variables, the effects of salinity treatments (control, EC 3, EC 5, and EC 7), inoculation (inoculated or non-inoculated), and their interaction were evaluated using two-way ANOVA, with salinity and inoculation as fixed factors. When significant salinity and inoculation interactions were present, inoculated and non-inoculated plants were analyzed separately to evaluate salinity responses within each group. Significant differences among means were determined using Tukey’s HSD post hoc test (*p* = 0.05). For experiments evaluating the effects of salinity on plant growth and BST, and for EC of water leachate and soil, data were analyzed by one-way analysis of variance (ANOVA) with treatment as the fixed effect. Means were compared using Tukey’s HSD post hoc test at a significance level of 0.05. Pairwise comparisons of plant fresh shoot weight between inoculated and non-inoculated plants were performed using independent Student’s t-tests within each salinity treatment. For antibacterial activity of salinity treatments against *X. perforans* in vitro and in planta, data were analyzed using a linear mixed model (PROC GLIMMIX), with treatment rate, time, and their interaction as fixed effects, and experiment as a random effect. An AR(1) covariance structure was used to account for repeated measurements over time. For leaf gas exchange measurements, data were sorted by week and inoculation. Leaf gas exchange measurements were analyzed separately by week and inoculation status. For each week and inoculation treatment, a one-way ANOVA was performed with salinity treatment as the fixed factor and net CO_2_ assimilation (*A*), transpiration (*E*), and stomatal conductance (*g_s_*) as response variables. Means were compared using the Waller–Duncan k-ratio test (*p* = 0.05). For the taste panel, total fruit yield was expressed as the total fruit mass harvested per treatment; therefore, fruit from all replicates was pooled, and no statistical analysis was performed. Flavor attributes (appearance, aroma, texture, etc.), as well as osmolality, osmotic potential and osmotic adjustment were analyzed using one-way ANOVA with treatment as the fixed effect. Post hoc comparisons were conducted using Tukey’s HSD at a significance level of 0.05. All error values are based on standard deviation.

## 5. Conclusions

In conclusion, this study provides valuable insights into how salinity affects tomato plants and BST development. Pre-exposure of tomato plants to salt (NaCl) treatments significantly reduced BST severity, likely through physiological effects such as stomatal closure, altered hormone signaling, and the activation of abiotic stress pathways. These findings highlight a trade-off associated with moderate salinity, where growth reductions may occur alongside enhanced disease reduction and improved fruit flavor. Under controlled conditions, moderate salt treatment shows potential for reducing BST, but its applicability in the field or commercial greenhouses and its overall impacts on yield require further validation.

## Figures and Tables

**Table 1:** Effect of salinity and inoculation with *X. perforans* on tomato plant growth and disease severity in greenhouse.

	Inoculated ^a^					Non-Inoculated ^a^	
Treatment	Height ^b ^(cm)	Weight ^c ^(g)	DS ^d ^(%)	AUDPC ^e^		Height ^b ^(cm)	Weight ^c ^(g)
Control	79.68 ± 2.67 a	63.17 ± 6.68 a	65.28 ± 17.78 a	248.9 ± 61.33 a		75.14 ± 7.17 a	95.6 ± 5.57 a
EC 3	79.22 ± 2.97 a	70.63 ± 8.28 a	13.72 ± 6.51 b	38.5 ± 17.80 b		74.5 ± 10.80 a	79.26 ± 4.51 b
EC 5	74.12 ± 5.92 ab	59.33 ± 8.56 ab	8.28 ± 4.26 b	22.8 ± 11.10 b		74.04 ± 5.24 a	73.52 ± 6.02 b
EC 7	70.12 ± 1.55 b	48.62 ± 3.76 b	3.39 ± 1.81 b	8.5 ± 4.52 b		71.60 ± 3.42 a	71.08 ± 5.30 b

^a^ Tomato cultivar = FL 47. Inoculated = tomato plants inoculated with QL strain of *X. perforans*. Non-Inoculated = Tomato plants not exposed to the pathogen. ^b^ Final tomato plant height measured from the soil surface to the shoot apex (cm). ^c^ Final fresh shoot weight measured by cutting at stem base (at the soil surface) and weighing (g). ^d^ DS = the average percentage of bacterial spot disease severity of three leaves at Day 11. ^e^ The area under the disease progress curve (AUDPC) was calculated based on disease severity ratings recorded throughout the experiment. Statistical analysis was done by ANOVA and means were separated using Tukey’s post-hoc test at *p* = 0.05. Values represent the means of six replicates (mean ± standard deviation [STDEV], *n* = 6). Different letters within columns indicate statistically significant differences among treatments.

**Table 2:** Electrical conductivity (EC) of water leachate and soil samples under salinity treatments.

	Water Leachate ^a^		Soil ^b^
Treatment	Beg. of Exp.	End of Exp.	End of Exp.
Control	1.64 ± 0.21 a	0.84 ± 0.04 d	0.24 ± 0.13 c
EC 3	1.89 ± 0.79 a	8.57 ± 1.51 c	2.33 ± 0.70 b
EC 5	1.67 ± 0.53 a	11.16 ± 0.78 b	4.51 ± 1.06 a
EC 7	1.99 ± 0.18 a	17.18 ± 0.40 a	5.08 ± 0.48 a

^a^ Electrical conductivity (EC, measured in dS m^−1^) of water leachate from pots treated with saline water at the beginning (beg.) and end of the experiment. ^b^ Electrical conductivity (EC, measured in dS m^−1^) of soil samples collected to evaluate salt accumulation in the soil at the end of the experiment. Statistical analysis was done by ANOVA followed by Tukey’s post-hoc test at *p* = 0.05. Values represent the means of three replicates (mean ± STDEV, *n* = 3). Different letters within columns indicate statistically significant differences among treatments.

**Table 3:** Osmotic adjustments of salt-treated tomato plants.

Treatment	Osm ^a^ (mmol/kg)	Ψs100 ^b^ (Mpa)	OA ^c^ (Mpa)
Control	0.32 ± 0.39 a	−0.80 ± 0.10 a	−
EC 3	0.34 ± 0.04 a	−0.85 ± 0.09 a	0.06 ± 0.07 a
EC 5	0.35 ± 0.046 a	−0.86 ± 0.11 a	0.06 ± 0.12 a
EC 7	0.36 ± 0.046 a	−0.89 ± 0.11 a	0.10 ± 0.12 a

^a^ Osmolality (Osm) = concentration of osmotically active dissolved particles in sap extracted from leaflets. ^b^ Ψs100 = osmotic potential at full turgidity. ^c^ Osmotic adjustment (OA) = difference in osmotic potential at full turgor between control and salt-treated plants. OA = Ψo (control treatment) 100 − Ψo (stressed treatment) 100. Statistical analysis was performed by ANOVA followed by Tukey’s post hoc test; same letters within columns indicate statistically no significant differences among treatments (*p* = 0.05). Values represent the means of twelve replicates (mean ± STDEV, *n* = 12).

## Data Availability

This manuscript includes all data analyzed during this project. Further inquiries can be directed to the corresponding author.

## Supplementary Materials

The following supporting information can be downloaded at: https://www.mdpi.com/article/10.3390/plants15162457/s1, Table S1: ANOVA *p*-values for the effects of salt treatment, inoculation, and their interaction on plant growth; Table S2: *p*-values of pairwise comparisons between inoculated and non-inoculated plants under each salinity treatment based on independent-samples t-tests; Table S3: ANOVA *p*-values for the effects of salt treatment, inoculation, and their interaction on gas exchange values; Table S4: Summary of RNA-seq data quality metrics and read alignment statistics for all samples Table S5: Effect of salinity on bacterial spot of tomato plants in a greenhouse; from RNA-seq experiment; Table S6: Electrical conductivity (EC) of water leachate and soil samples under salinity treatments from RNA-seq experiments; Table S7: RNA-seq sample collection timeline and experimental design; Table S8: Top enriched GO terms associated with DEGs between inoculated EC 7 and inoculated CK plants across timepoints; Table S9: Top enriched KEGG pathways associated with DEGs between inoculated EC 7 and inoculated CK plants across timepoints; Table S10: Raw gene count data; Figure S1: Evaluation form completed by all participants in the taste panel to assess quality of tomatoes grown under salinity treatments.
