# Supplementary material for: Effects of Salinity on Bacterial Spot Disease, Physiology, Growth, Fruit Quality, and Transcriptomic Responses in Tomato Plants
Source: Plants (Basel). 2026 Aug 13;15(16):2457. doi: 10.3390/plants15162457 (PMC13516753; doi:10.3390/plants15162457)
Supplement: Supplementary file 1 [file plants-15-02457-s001.zip › Plants supplemental Figures.pdf]

Tomato Taste Evaluation Sheet

Panelist Name: ___________
Date: ___________
Instructions:

- You will be tasting four tomato samples labeled **A**, **B**, **C**, and **D** (in randomized order).
- For each sample, rate the following attributes on a scale of **1 (Poor)** to **5 (Excellent)**.
- Please focus on each attribute individually and provide your honest feedback.

Attribute	Sample A	Sample B	Sample C	Sample D
**Appearance**	☐ 1 ☐ 2 ☐ 3 ☐ 4 ☐ 5	☐ 1 ☐ 2 ☐ 3 ☐ 4 ☐ 5	☐ 1 ☐ 2 ☐ 3 ☐ 4 ☐ 5	☐ 1 ☐ 2 ☐ 3 ☐ 4 ☐ 5
**Aroma**	☐ 1 ☐ 2 ☐ 3 ☐ 4 ☐ 5	☐ 1 ☐ 2 ☐ 3 ☐ 4 ☐ 5	☐ 1 ☐ 2 ☐ 3 ☐ 4 ☐ 5	☐ 1 ☐ 2 ☐ 3 ☐ 4 ☐ 5
**Texture**	☐ 1 ☐ 2 ☐ 3 ☐ 4 ☐ 5	☐ 1 ☐ 2 ☐ 3 ☐ 4 ☐ 5	☐ 1 ☐ 2 ☐ 3 ☐ 4 ☐ 5	☐ 1 ☐ 2 ☐ 3 ☐ 4 ☐ 5
**Sweetness**	☐ 1 ☐ 2 ☐ 3 ☐ 4 ☐ 5	☐ 1 ☐ 2 ☐ 3 ☐ 4 ☐ 5	☐ 1 ☐ 2 ☐ 3 ☐ 4 ☐ 5	☐ 1 ☐ 2 ☐ 3 ☐ 4 ☐ 5
**Acidity**	☐ 1 ☐ 2 ☐ 3 ☐ 4 ☐ 5	☐ 1 ☐ 2 ☐ 3 ☐ 4 ☐ 5	☐ 1 ☐ 2 ☐ 3 ☐ 4 ☐ 5	☐ 1 ☐ 2 ☐ 3 ☐ 4 ☐ 5
**Taste**	☐ 1 ☐ 2 ☐ 3 ☐ 4 ☐ 5	☐ 1 ☐ 2 ☐ 3 ☐ 4 ☐ 5	☐ 1 ☐ 2 ☐ 3 ☐ 4 ☐ 5	☐ 1 ☐ 2 ☐ 3 ☐ 4 ☐ 5
**Overall**	☐ 1 ☐ 2 ☐ 3 ☐ 4 ☐ 5	☐ 1 ☐ 2 ☐ 3 ☐ 4 ☐ 5	☐ 1 ☐ 2 ☐ 3 ☐ 4 ☐ 5	☐ 1 ☐ 2 ☐ 3 ☐ 4 ☐ 5

Ranking of Samples:
Please rank the samples from your most preferred to least preferred:
1st choice: _______
2nd choice: _______
3rd choice: _______
4th choice: _______
Additional Comments: (Any notes, preferences, or specific feedback you would like to add**)**

Figure S1. Evaluation form completed by all participants in the taste panel to assess quality of tomatoes grown under salinity treatment
